# Retraction: *In vitro* and *in vivo* plant growth promoting activities and DNA fingerprinting of antagonistic endophytic actinomycetes associates with medicinal plants

**DOI:** 10.1371/journal.pone.0341487

**Published:** 2026-01-26

**Authors:** The PLOS One Editors

Following the publication of this article [1], concerns were raised about Fig 2B. Specifically, the bands in lanes numbered 5, 21 and 24–26 appear more similar to the bands in lanes numbered 37 and 39–42, respectively, than would be expected from independent samples.

In light of the above concerns that question the reliability and integrity of the results presented in [1], the *PLOS One* Editors retract this article.

All authors did not agree with the retraction.
